# Novel pyroptosis-associated genes signature for predicting the prognosis of sarcoma and validation

**DOI:** 10.1042/BSR20221053

**Published:** 2022-12-09

**Authors:** Hao Wen, Dandan Guo, Zhenguo Zhao, Xin Xin, Qi Shi, Jiachen Cao, Lingxie Song, Yuliang Jiang, Chunxia Liu, Feng Li

**Affiliations:** 1 Department of Pathology and Key Laboratory for Xinjiang Endemic and Ethnic Diseases, The First Affiliated Hospital, Shihezi University School of Medicine, Shihezi 832002, China; 2 Medical Research Center and Department of Pathology, Beijing Institute of Respiratory Medicine and Beijing Chao-Yang Hospital, Capital Medical University, Beijing 100020, China; 3 Department of Orthopaedics, National Cancer Center/National Clinical Research Center for Cancer/Cancer Hospital, Chinese Academy of Medical Sciences and Peking Union Medical College, Beijing, China; 4 Department of Oncology, Beijing Chao-Yang Hospital, Capital Medical University, Beijing 100020, China; 5 Department of Pathology, The Second Affiliated Hospital of Guangzhou Medical University, Guangzhou 510260, China

**Keywords:** biomarkers, pyroptosis, sacoma

## Abstract

Background: Sarcoma is a rare mesenchymal malignant tumor. Recently, pyroptosis has been reported to be a mode of programmed cell death. Nonetheless, levels of pyroptosis-associated genes in sarcoma and its relevance to prognostic outcomes are yet to be elucidated. Results: Sarcoma cases were classified into two subtypes with regards to differentially expressed genes. We established a profile composed of seven genes and classified the sarcoma patients into low- and high-risk groups through least absolute shrinkage and selection operator Cox regression. Survival rate of low-risk sarcoma patients was markedly higher, relative to high-risk group (*P*<0.001). In combination with clinical features, the risk score was established to be an independent predictive factor for OS of sarcoma patients. Chemotherapeutic drug sensitivity response analysis found 65 drugs with higher drug sensitivity in low-risk, than in high-risk group and 14 drugs with higher drug sensitivity in the high-risk patient group, compared with low-risk patient group. In addition, functional enrichment, pathway and gene mutation of the two modules were analyzed. Finally, we used qRT-PCR to detect the expression of seven pyroptosis-related genes in tumor cells, and human skeletal muscle cells, compared with human skeletal muscle cells, PODXL2, LRRC17, GABRA3, SCUBE3 and RFLNB genes show high expression levels in tumor cells, while IGHG2 and hepatic leukemia factor show low expression levels in tumor cells. Conclusions: Our research suggest that pyroptosis is closely associated with sarcoma, and these findings confirm that pyroptosis-associated seven genes have a critical role in sarcoma and are potential prognostic factors for sarcoma.

## Introduction

Sarcoma is a kind of rare malignancy of mesenchymal tissues [1]. The disease tends to occur in mesenchymal tissue, which includes bones, fat, joints and muscles [2]. Sarcomas are grouped into two, osteosarcoma and soft-tissue sarcoma [3]. Sarcoma pathogenesis is associated with fusion genes, chromosome mutations and other factors, but its underlying pathogenic mechanismis are still unclear. Although the source, histological and molecular markers of sarcomas differ, their common feature is poor disease prognosis [4]. At present, the principal treatments are radiotherapy and chemotherapy in the clinical, and no targeted drugs have been proved to be effective [5]. Therefore, it is urgent to further study the molecular mechanism and signaling pathway of sarcoma.

Programmed cell death mechanisms, such as apoptosis, necroptosis and pyroptosis have distinct inflammatory outcomes. Among them, the inflammation of pyrotopia is more severe compared with apoptosis [6]. Pyroptosis is a programmed cell death also called inflammatory necrosis [7]. Pyrocytes are featured with cell swellings and various vesicular projections. Under electron microscopy, pyrophosis cells first appear in form of many vesicles. Upon the formation of these vesicles, they form holes in cell membranes, leading to rupture and release of their contents [8]. Gasdermin family is the major genes of pyroptosis including gasdermin-A, -E as well as pejvakin (DFNB59 or PJVK) [9]. The gasdermin protein family can be cleaved and polymerized, resulting in the splitting of N- and C-terminal linking domains and the release of activated N-terminal regions. These released regions bind membrane lipids, cardiolipin as well as phosphatidylinositol and are located in cell membrane pores [10,11]. The cell gasdermin protein family in the cell membranes are gradually released from pores of 10–20 nm, and the cell components are gradually released via membrane pores, leading to enhanced inflammatory reactions. The cells slowly flattened and produced 1–5 μm of apoptotic vesicle-like processes (burned vesicles). The cells slowly expanded to rupture of plasma membrane, which was characterized by nuclear condensation as well as chromatin DNA fragmentation [12,13]. Initially, pyrotopia was established to be a crucial pathomechanism in fighting infection and is also involved in tumor occurrence. Proinflammatory cytokines, inflammatory vesicles and gasdermin proteins are reported to be key components of pyroptosis and are relevant to tumor genesis, invasion and metastasis [14]. Dupaul-Chicoine et al. reported that when the inflammatory vesicle-associated genes (CASP1 and NLRP3) were knocked out in transgenic mice, they were predisposed to colon cancer development, relative to mice with the wild-type gene [15]. In addition, unlike apoptosis, various risk-related signaling molecules as well as cytokines are activated and thereafter released when pyroptosis occurs, accompanied by severe inflammatory reactions and immune system activation [16]. The strong proinflammatory result of pyroptosis is correlated with tumor immune microenvironment regulation. Expression deficiency of GSDMD was followed by a marked decrease in numbers and activities of CD8+T lymphocytes [17]. The crucial role of pyrophosis in NK cell antitumor function was also demonstrated in a recent study [18].

According to the literature, we conclude that pyroptosis is vital in tumor occurrence as well as anticancer processes. However, its precise roles in sarcomas are rarely studied. Therefore, this was a systematic analysis to assess the association between sarcomas and levels of pyroptosis-associated genes, and to provide new therapeutic targets and options for sarcomas.

## Materials and methods

### SARC datasets and preprocessing

Analyses of Cancer Genome Atlas (*TCGA*) datasets were downloaded in *UCSC Xena* data browser (*https*://*xenabrowser.net*). We converted the number of fragments per kilobase of non-overlapped exon per million fragments mapped (FPKM) to Gene expression levels which were then quantified as transcripts per million reads (TPM) values. The R software (version 3.6.1) and a collection of R/Bioconductor packages were used for analyses.

### Clustering genes of pyroptosis-associated genes

Thirty-three pyroptosis-associated genes were retrieved from prior reviews [19–22]. Clustering of sarcoma transcriptome profiles was performed using NMF implemented in ‘*nmf*’ in R (version NMF_0.20.5). R packages (PCA methods) were used for PCA analyses.

### Building and verifying the pyroptosis-related gene prognostic model

‘Limma’ in R was used for identifying differentially expressed genes (DEGs) with *P*<0.05. Notation of DEGs was as: *P<0.05, **P<0.01 and ***P<0.001. To evaluate the prognostic significance of pyroptosis-associated genes, we used Cox regression analyses to determine the association between survival status and every gene in TCGA cohort. To preclude omissions, 0.2 was the cut-off *P*-value, random survival forest analysis was used to filter variable. LassoCox regression model (‘GLmnet’ in R) was used to identify the candidate genes and establish prognosis models. Finally, seven genes with their coefficients were preserved, and the penalty parameter (λ) was determined according to the minimum criterion. After standardizing TCGA expression data sets (applying the ‘scale’ function in R), calculate the risk score: Risk score = $\sum_{i}^{7}Xi\times Yi$ (*Y*: levels of gene expressions, *X*: coefficients). TCGA sarcoma patients were assigned into low-risk and high-risk subgroups based on median risk scores. Circos plot was created using modified functions from the R package ‘RCircos’. Kaplan–Meier analyses were used for comparisons of OS time between the two subgroups. Receiver operating characteristic (ROC) curve analysis was performed for 1-5 years using ‘Survival’, ‘Survivor’ and ‘timeROC’ R packages.

### GDSC cell line drug response data

The GDSC large-scale composite reaction screening dataset was obtained [23], which included 990 human cancer cell lines from 25 cell lineages and 255 chemical compounds. The 255 compounds came had a variety of sources, such as candidates for clinical drugs, FDA-permitted drugs, and previously reported chemical sensitivity analysis assays. For drug sensitivity quantification, IC50 from GDSC were used [23]. In order to determine approved to treat various varieties of cancer drugs, a manual search of the usages of these drugs was performed in Wikipedia and the NCI database (https://www.cancer.gov/about-cancer/treatment/drugs). The drugs for every tumor type are shown in. We also downloaded the somatic mutation profiles and tissues of origin for the 990 cancer cell lines [24]. We applied the propagation algorithm based on ESP to divide every cancer cell line into two clusters, using only the sub-network data. We then used rank-based Wilcoxon-type statistics for comparisons of differences in drug reactions between the clusters.

### Immunocorrelation analyses and gene expressions

Meanwhile, we compared CIBERSORT [25,26], ESTIMATE [27], MCPcounter [28], single-sample gene set enrichment analysis (ssGSEA) [29] as well as TIMER [30] algorithms, to evaluate cell constituents or cellular immune reactions between high- and low-risk groups with a basis on the characteristics of pyroptosis-associated genes. Heatmaps were employed to reveal differences in immune reactions under various algorithms. Moreover, ssGSEA was used for quantifying subsets of tumor-infiltrating immune cells between the groups and for immune function evaluation. Immune checkpoints were acquired from literature. Cluster Profiler in R was used for gene set enrichment analysis (GSEA).

### Function and pathway enrichment analyses

GO offers regulated and structured vocabularies that model cell components (CC), biological process (BP) and molecular function (MF) [31]. KEGG is a commonly used pathway analysis method, which contains 16 major databases, assigned into systematic information, chemical and genomics information [32]. We combined GO and KEGG analyses to conduct DEGs. The cut-off was p <0.05. All gene sets related to GO and KEGG were downloaded from MSigDB database.

### Analysis of tumor mutation in sarcoma

As a quantifiable biomarker, tumor mutation burden (TMB) can be used to reflect the counts of mutations contained in cancer cells. In our study, tumor mutational burden of sarcoma patients was calculated as follows: TMB = *S_n_* × 1,000,000/*n* (*S_n_* is the absolute somatic mutation value, and *n* denotes the counts of exon base coverage depth ≥ 100×) [33].

### Cell lines and culture conditions

Human skeletal muscle cells (HSKMC) and Rhabdomyosarcoma cells (RH30) were purchased from American type culture collection (ATCC, U.S.A.). HSKMC were cultured in Mesenchymal Stem Cell Basal Medium with Primary Skeletal Muscle Cell Growth Kit (ATCC, U.S.A.) at 37°C in a humidified atmosphere containing 5% CO2. RH30 were cultured in were cultured in RPMI 1640 medium with 10% fetal bovine serum (FBS) (Gibco, Grand Island, NY, U.S.A.) and 1% penicillin-streptomycin at 37 °C in a humidified atmosphere containing 5% CO_2_. Synovial sarcoma cells (SW982) were purchased from FUXIANG Biotechnology (Shanghai, China). SW982 were cultured in were cultured in Dulbecco’s Modified Eagle Medium with 10% fetal bovine serum (FBS) (Gibco, Grand Island, NY, U.S.A.) and 1% penicillin-streptomycin at 37 °C in a humidified atmosphere containing 5% CO_2_.

### RNA extraction and real-time quantitative PCR (qRT-PCR)

Total RNA was extracted using Trizol (Invitrogen) according to manufacturer’s protocol. One microgram of total RNA was used to synthesize cDNA using random hexamers and the Superscript III First-Strand Synthesis System for RT-PCR (Invitrogen). The real-time PCR was performed using the Fast SYBR Green Master Mix (Applied Biosystems) and run on a 7500 Fast Real-Time PCR System machine (Applied Biosystems) in the fast mode. The relative number of mRNAs was calculated by using the ΔΔ*Ct* (*Ct*, threshold cycle) method.

Primers sequence are as follows:

**Table:** 

IGHG2 (94 bp):
Forward 5′-AGGGACAACTCCGCAAACAC-3′
Reverse 5′-TCCCCGAATGTGCTTTCGC-3′
PODXL2 (144 bp):
Forward 5′-CTCCCTGCTAGACCTCCTG-3′
Reverse 5′-TGCAGAATCCGAGACTCTTCAT-3′
LRRC17 (177 bp):
Forward 5′-AGAAGCCGAGTGAATCATGGC-3′
Reverse 5′-GTGCAGCAAATCCTGAGGC-3′
GABRA3 (145 bp):
Forward 5′-CAAGGGGAATCAAGACGACAA-3′
Reverse 5′-CGTCCAGAAGACGATCCAAGAT-3′
SCUBE3 (135 bp):
Forward 5′-CAGAACACCCCGAGGTCATAC-3′
Reverse 5′-GCCAGGGATGTTGACACAGTC-3′
HLF (103 bp):
Forward 5′-CTGGGGCCTACCTTATGGGA-3′
Reverse 5′-GGGGAATGCCATTTTCTGACA-3′
RFLNB (188 bp):
Forward 5′-AGGCACTTCATCGACGACG-3′
Reverse 5′-TAGACGGCCTTGGGGTACTT-3′
GAPDH (197 bp)
Forward 5′-GGAGCGAGATCCCTCCAAAAT-3′
Reverse 5′-GGCTGTTGTCATACTTCTCATGG-3′

### Statistical analyses

Verification of normality of variables was done by the Shapiro–Wilk normality test. We use the *T*-test to compare differences between two sets of normally distributed variables. Comparisons of non-normally distributed variables was done by Wilcoxon signed rank test. Pearson correlation coefficient is used to measure similarity, and Pearson correlation distance is used to estimate dissimilarity. ‘ggplot2’ in R was used for plot generation, while R package ‘randomForest’, in R, version 3.6.1 was used to build the Random Forest models. Survival-ROC curves were applied with ‘timeROC’ the package. Kaplan–Meier was applied to estimate survival probability and generation of survival curves. Statistical differences in survival data were evaluated by the log-rank test. Analysis and survival curves were by ‘survminer’ and survival in R. Heatmap was generated using pheatmap (v1.0.12). R version 3.6.1 (https://www.r-project.org/) was used for analyses. All the tests were two-sided with *P*<0.05 denoting significance. Mutated genes were assessed by the R package maftools v2.2.10. GSEA and enrichment analysis are implemented by R package clusterProfiler.

## Result

### Classification of tumors based on pyroptosis-associated genes

**Figure 1:**
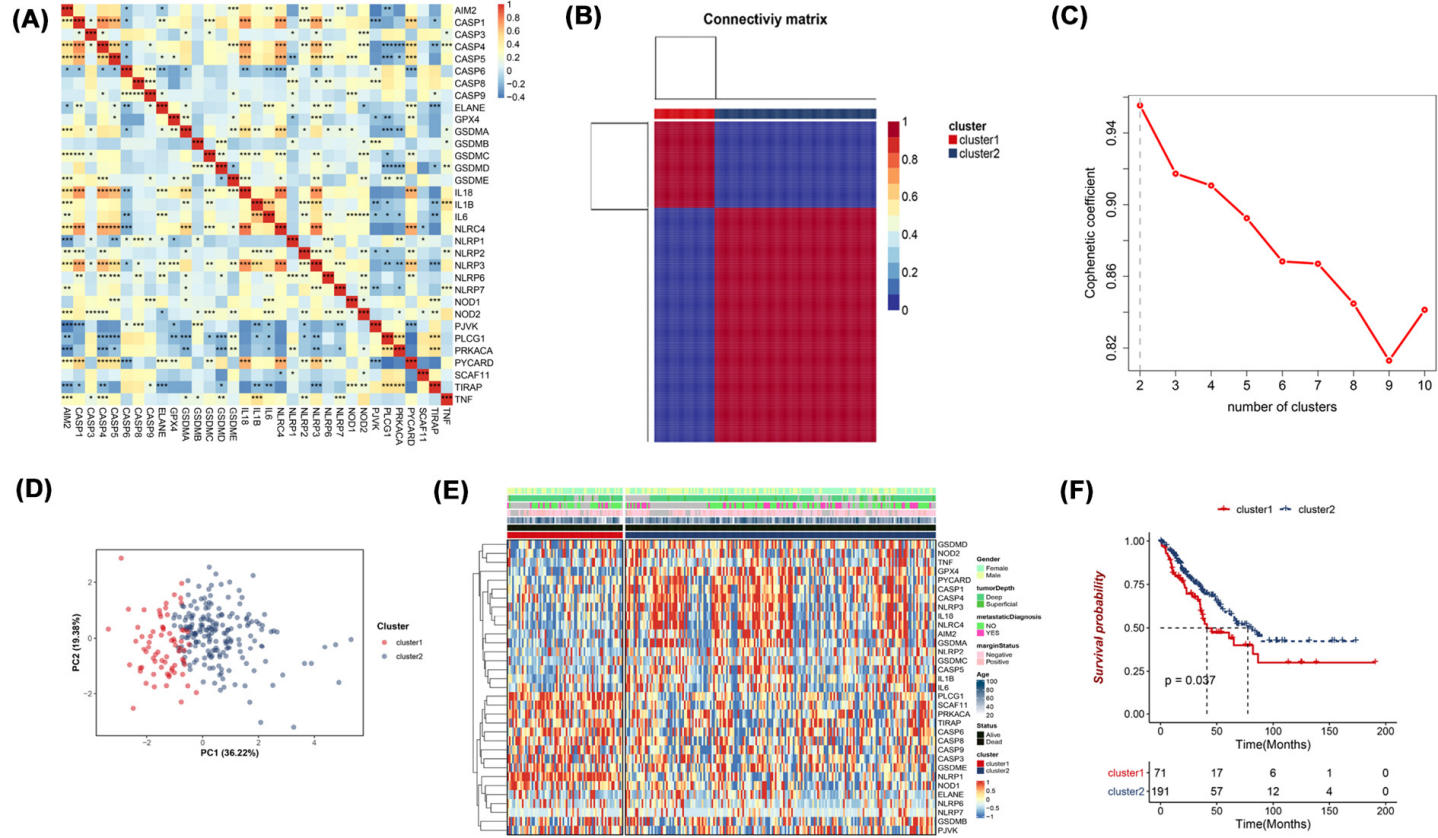
Tumor classification based on the pyroptosis-related DEGs (**A**) Pairwise correlations among the levels of 33 pyroptosis-associated genes in sarcoma. (**B**) About 262 Sarcoma patients assigned into two clusters based on consensus clustering matrix (*k* = 2). (**C**) When the clustering variable (*k*) was increased from 2 to 10, (*k* = 2) is the best clustering. (**D**) The PCA plot for sarcoma patients, which was based on the pyroptosis-related genes. (**E**) Heatmap and clinic-pathologic features of the two clusters, as classified by the DEGs. (**F**) Kaplan–Meier OS curves of the two clusters.

To evaluate associations among pyroptosis-associated genes, we calculated pairwise correlations among the expression of 33 pyroptosis-associated genes in sarcoma, which showed that pairs of these genes are commonly linked, and the negative correlations were more frequent than positive correlations (Figure 1A). And then, NMF algorithm was used to cluster sarcoma samples in the TCGA dataset. To investigate the association between levels of 33 pyroptosis-associated genes and sarcoma subtypes, consensus cluster analysis was performed on 262 sarcoma patients in the TCGA cohort. We performed NMF unsupervised clustering of pyroptosis-related genes in classes 2–10 and found that the best clustering was achieved when clustering two classes, when the clustering variable (*k*) was increased from 2 to 10, it was established that at *k* = 2, intragroup and intergroup associations were highest and low, respectively, implying that the 262 sarcoma patients can be assigned into two clusters based on the 33 pyroptosis-associated genes (Figure 1B,C). Then, principal component analysis (PCA) was used to verify the clustering situation. PCA analysis indicated two clusters are two distinct groups (Figure 1D). The gene expression profiles as well as clinical characteristics, such as age, gender, tumor depth, metastatic diagnosis. Margin status are displayed in the heatmap. Differences in clinical characteristics between the clusters were minimal (Figure 1E). In an assessment of overall survival rate in the two clusters, patients between the two clusters have a significantly different overall survival rate (*P*=0.037, Figure 1F).

### Establishment of prognostic gene models in TCGA

**Figure 2:**
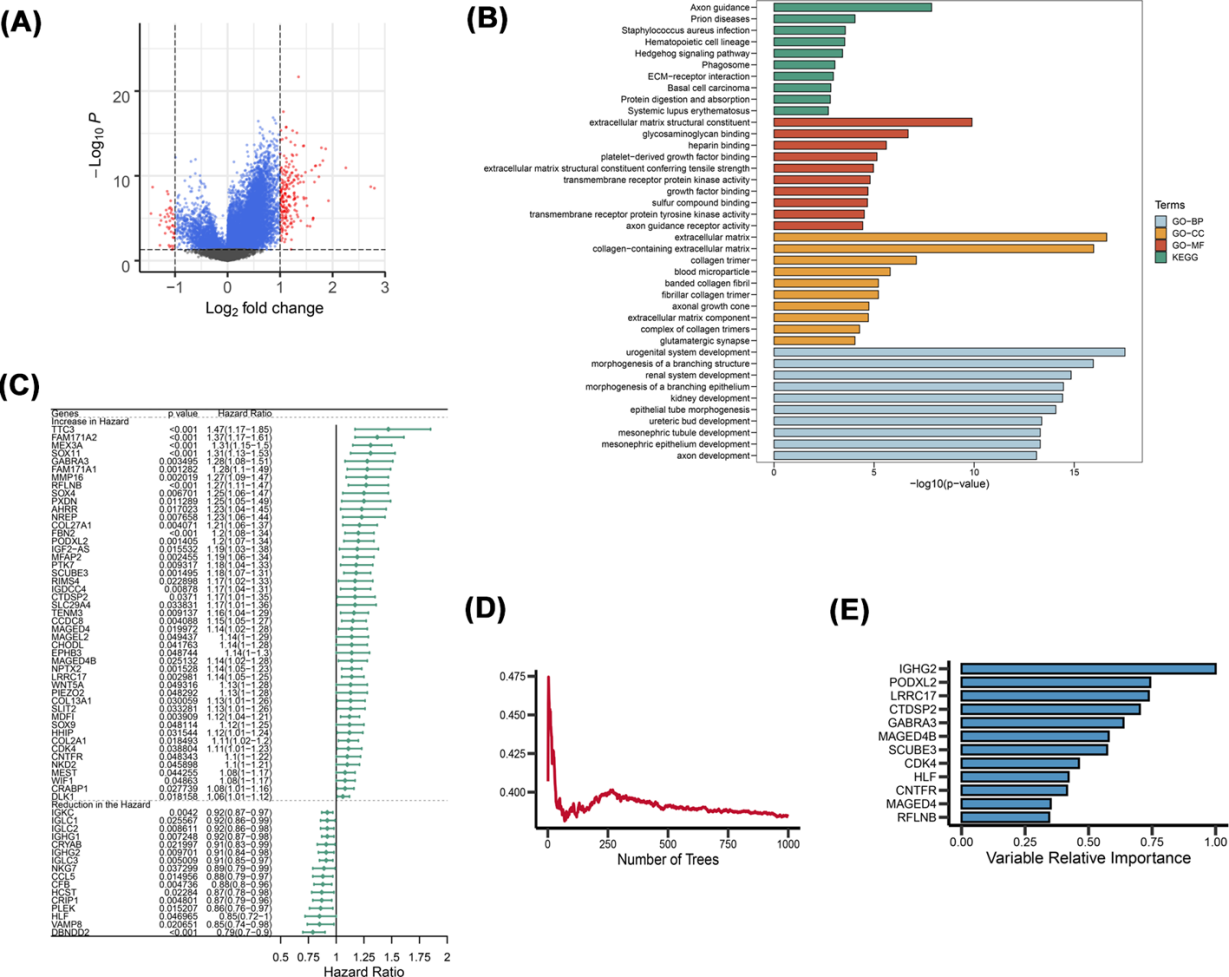
The screening and functional enrichment analyses of the DEGs (**A**) Volcano map of DEGs. (**B**) GO and pathway analyses of DEGs. (**C**) Sixty-three were established by univariate Cox Regression analyses for dimensionality reduction. (**D**) Error rate for the data as a function of the classification tree. (**E**) The importance values for 12 the predictors.

**Figure 3:**
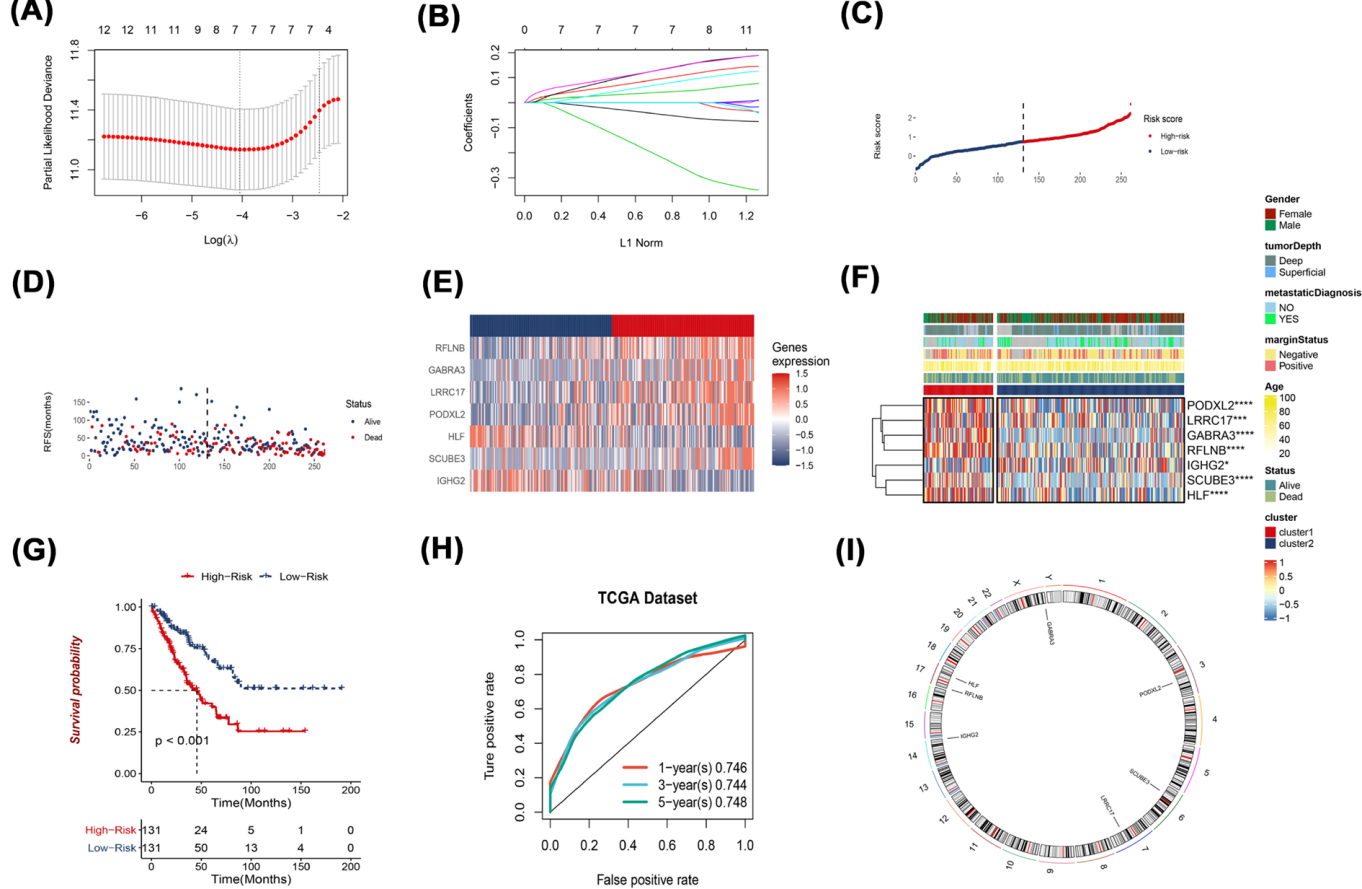
Creation of the risk signature in TCGA cohort (**A**) Cross-validation for tuning parameter selections in LASSO regression. (**B**) LASSO regression of seven genes. (**C–E**) Risk score distribution, survival status, and expressions of seven prognostic pyroptosis-related genes in sarcoma. (**F**) Heatmap of connections between clinic-pathologic characteristics and seven genes. (**G,H**) Overall survival curves for high-/low-risk group sarcoma patients and ROC curve for evaluating the predictive value. (**I**) Circos plot shows the chromosomal distribution of the seven genes.

DEG analysis was performed to compare the differential gene expressions of the two clusters. The volcano map shows that 246 genes have been analyzed (Figure 2A). In addition, KEGG pathway enrichment analyses and functional evaluations were done using the Enrichr database, including three types of GO analysis: BP, CC and MF. These genes were highly enriched in Axon guidance in BP. In terms of CC, they were enriched in extracellular matrix structural constituent. As for MF, these genes are mainly associated with extracellular matrix and collagen-containing extracellular matrix. KEEG enrichment showed that 246 DEGs were associated with urogenital system development and other aspects (Figure 2B). In order to obtain more accurate genes related to cell pyroptosis, we reduced DEG genes to 63 by univariate Cox regression analysis dimension reduction (Figure 2C), and then 12 genes (IGHG2, PODXL2, LRRC17, CTDSP2, GABRA3, MAGED4B, SCUBE3, CDK4, hepatic leukemia factor [HLF], CNTFR, MAGED4 and RFLNB) were obtained by random survival forest (Figure 2D,E). A signature of seven genes was established using the optimal λ value through LASSO Cox regression (Figure 3A,B). The risk score formula was: risk score = (-0.0588*IGHG2 exp.) + (0.1123*PODXL2 exp.) + (0.0476*LRRC17 exp.) + (0.0905*GABRA3 exp.) +(0.1529*SCUBE3 exp.) + (-0.271*HLF exp.) + (0.1519*RFLNB exp.). With regards to the median score as determined by the risk score formula, 262 patients were assigned into low- and high-risk sub-groups (Figure 3C). High-risk group patients were correlated with a high death rate and shorter survival times, relative to low-risk patient group (Figure 3D, on right side of dotted line). Among the seven genes (IGHG2, PODXL2, LRRC17, GABRA3, SCUBE3, HLF and RFLNB), the expression levels of PODXL2, LRRC17, GABRA3, RFLNB and SCUBE3 in high-risk group were evidently higher, relative to low-risk group. Expressions of IGHG2 as well as HLF were markedly low in high-risk group than in the low-risk group (Figure 3E). Subsequently, we analyzed the relationship between these seven genes and the two clusters, as well as the correlation between these seven genes and clinical indicators (Figure 3F), IGHG2, PODXL2, LRRC17, GABRA3, SCUBE3, HLF, and RFLNB were associated with gender, tumor depth, metastatic diagnosis, margin stage, age, and status. Notable differences in OS time were detected between low- and high-risk groups (*P*<0.001, Figure 3G), patients with in high-risk groups have significantly worse overall survival. Time-dependent ROC analysis was used to assess the models’ specificity and sensitivity. Notably, we the area under the ROC curve (AUC) for survival was respectively 0.746, 0.744, and 0.748 for 1, 3 and 5 years (Figure 3H). In addition, these seven genes, are distributed in randomly chromosome and shown in the Circos plot (Figure 3I).

### Prognostic significance of the risk model

**Figure 4:**
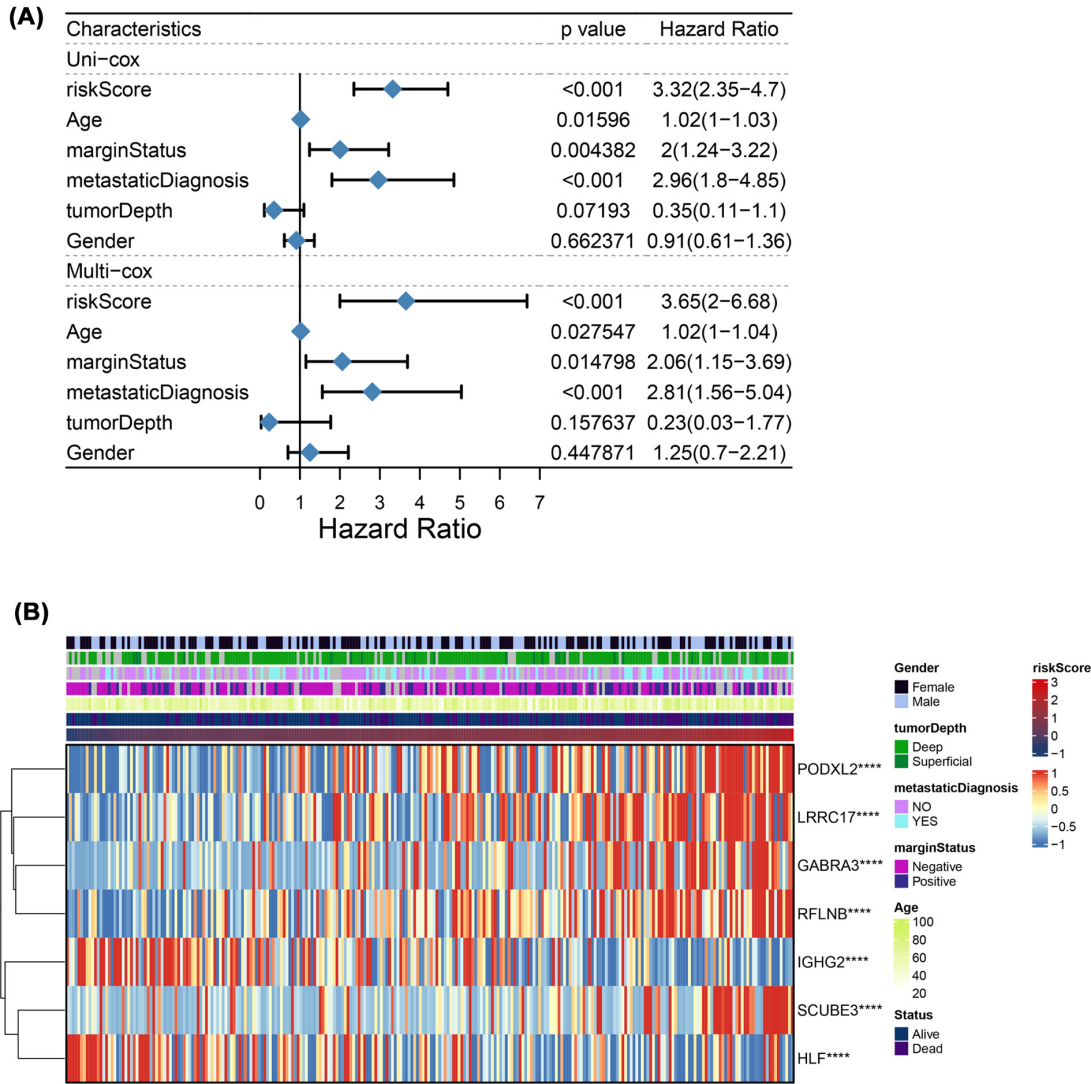
Univariate as well as multivariate Cox regression analyses of the risk score (**A**) Univariate and Multivariate analysis for the TCGA cohort. (**B**) Heatmap of connections between clinic-pathologic characteristics and risk groups.

The risk score was an independent factor that was a factor in predicting low survival by univariate Cox regression analyses in TCGA cohort (HR = 3.32, 95% CI: 2.35–4.7, Figure 4A). After adjustment for other con-founding factors, multivariate analysis implied that the risk score is a prognostic factor (HR = 3.65, 95% CI: 2–6.68, Figure 4A) for patients with Sarcoma in the cohort. Furthermore, the clinical characteristic heatmaps of the TCGA cohort are shown (Figure 4B) and found that PODXL2, LRRC17, GABRA3, SCUBE3 and RFLNB have positive correlation with risk score. On the contrary, IGHG2 and HLF have negative correlation with risk score.

### Identify drug candidates for risk models

**Figure 5:**
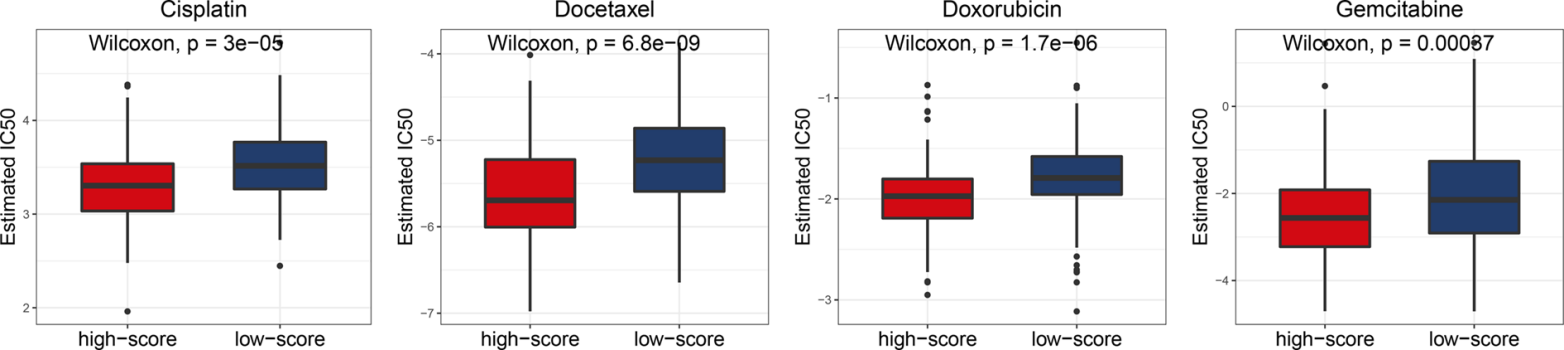
Chemotherapeutic response for risk models High-risk group patients were sensitive to chemotherapy with cisplatin, docetaxel, doxorubicin and gemcitabine.

We found that the IC50 values of 65 drugs in high-risk patient group were markedly low relative to those in low-risk patient group. At present, the main chemotherapy drugs for sarcoma include cisplatin, docetaxel, doxorubicin, gemcitabine and so on. IC50 values implying that high-risk group patients were sensitive to chemotherapy with cisplatin, docetaxel, doxorubicin and gemcitabine. Hence, these drugs may be more appropriate for high-risk patients. This may provide a new idea for clinical chemotherapy regimen (Figure 5). In addition, IC50 values of 14 drugs, including erlotinib, gefitinib, etc., were higher in high-risk group, relative to low-risk group, and the high-risk group may not be sensitive to the chemotherapy response of these 14 drugs. IC50 values of all 79 drugs are shown in Supplementary Figures.

### Different functional pathways analyses of the risk models

**Figure 6:**
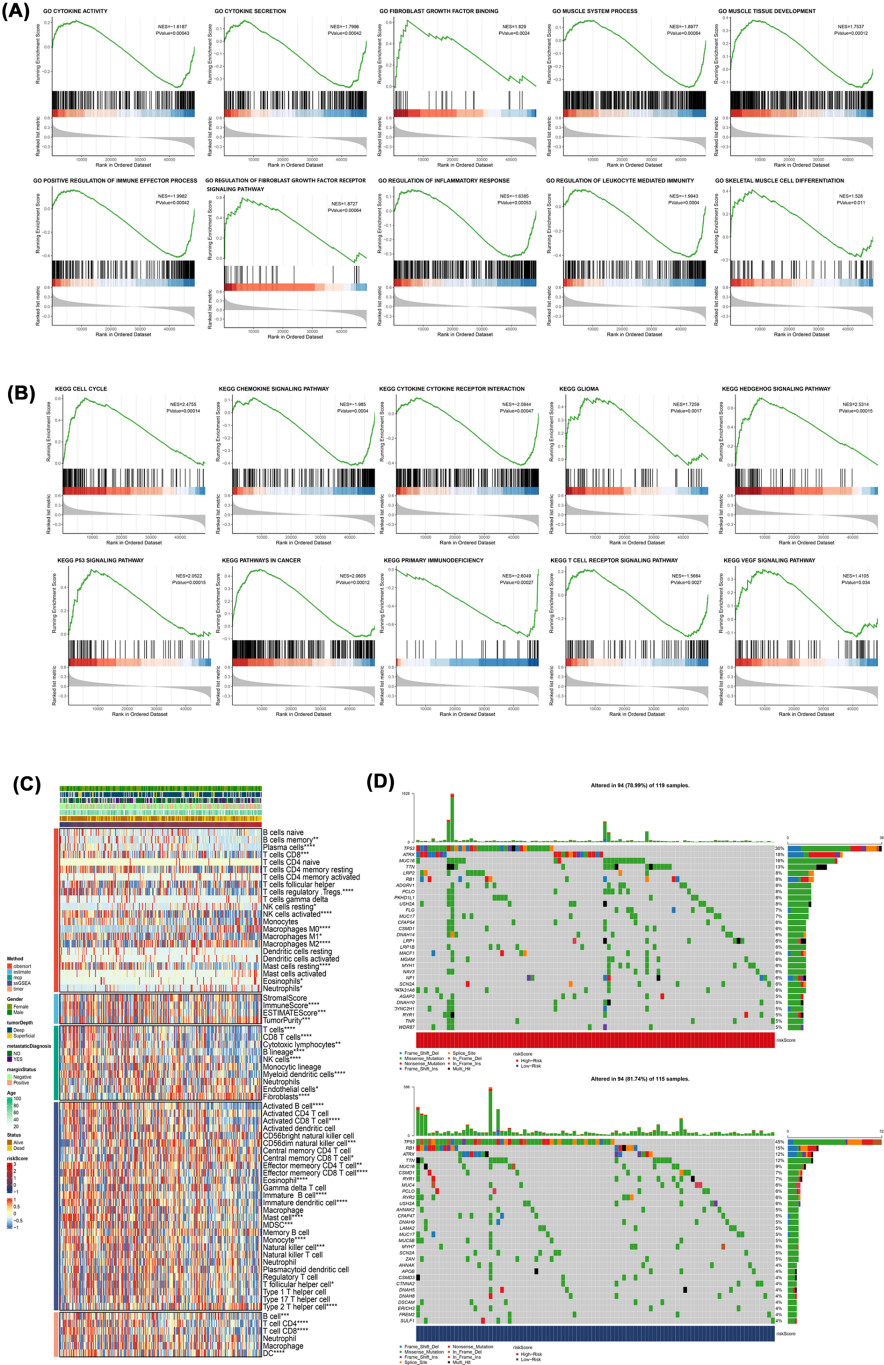
Functional analysis of risk models (**A**) GO enrichment of the risk models. (**B**) KEGG pathway enrichment of the risk models. (**C**) Immune response analysis of risk models. (**D**) Mutation analysis of risk models.

To explore the reasons for different sensitivity of drugs in high- and low-risk groups, we conducted the GSEA of our models. GO term analyses revealed that molecular function of high-risk patient group was significant enrichment in the cytokine activity, cytokine secretion, fibroblast growth factor binding, muscle system process, muscle tissue development, positive regulation of immune effector processes, fibroblast growth factor receptor signaling pathway regulation, inflammatory reaction regulation, regulation of leukocyte-mediated immunity and skeletal muscle cell differentiation (Figure 6A). KEGG pathway analyses also showed the pathways enrichment of high-risk group in cell cycle, such as chemokine signaling pathway, cytokine–cytokine receptor asssociations, glioma, hedgehog and p53 signaling pathways, pathways in cancer, T-cell receptor signaling pathway, primary immunodeficiency and VEGF signaling pathway (Figure 6B). To further dig into the reasons for the differences of sensitivity of drugs, we analyzed the different modules in terms of immunity and mutation. The immune responses heatmap is presented. CIBERSORT, TIMER, ssGSEA and MCP counter showed that the levels of infiltration of immune cells were usually lower in high-risk patient group, particularly CD8+ T cells. ESTIMATE revealed that at a higher risk score, tumor purity was higher, and the immune score was lower (Figure 6C). These results are consistent with previous analyses. Moreover, Oncoplot shows the top 30 mutated genes of low- and high-risk groups, among them, mutations of TP53, RB1, MUC16 and some other genes between the groups were markedly different. These differences in immune response and genetic mutation may account for the differences in sensitivity to chemotherapeutic drugs between high- and low-risk groups (Figure 6D).

### Validation of pyroptosis-associated genes expression by qRT-PCR

**Figure 7:**
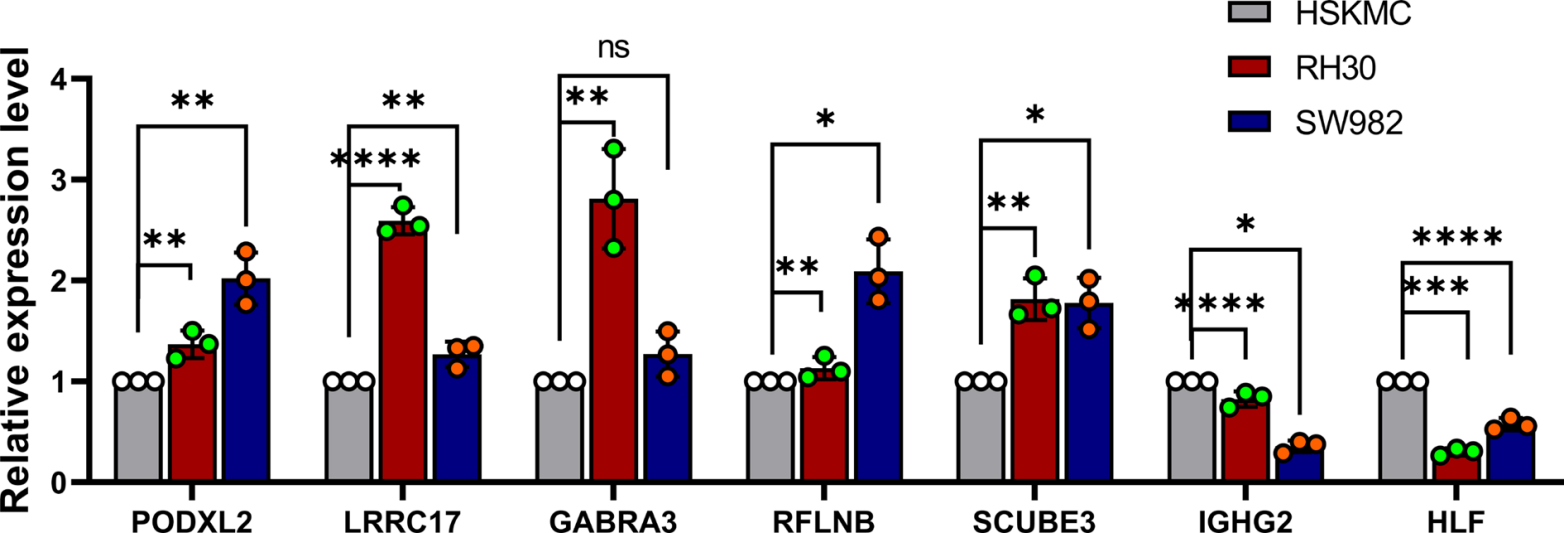
Validation of seven genes expression in RH30, SW982 and HSKMC by qRT-PCR Bars represent mean ± SEM (*n*=3), *P<0.05, **P<0.01, ***P<0.001, ****P<0.0001.

To validate the expression levels of seven pyroptosis-associated genes in sarcoma, we used qRT-PCR to verify RNA expression levels in rhabdomyosarcoma cell line— RH30, synovial sarcoma cell line—SW982, and human normal skeletal muscle cell—HSKMC. The results showed that compared with HSKMC, PODXL2, LRRC17, GABRA3, SCUBE3, and RFLNB were highly expressed in tumor cells, while IGHG2 and HLF were low expressed (Figure 7). This result is consistent with our previous data analysis.

## Discussion

Sarcoma is a rare tumor with local invasion and distant metastasis. The prognosis is poor. Histological biopsy is the most authoritative standard for the diagnosis of Sarcoma [34,35]. The main therapeutic methods are radiation therapy and surgery [36], and there are still no proven target drugs for sarcomas. Up to now, the mechanism of sarcomas is still poorly understood, which is the main obstacle to the development of specific targeted drugs.

Pyroptosis, a newly discovered mode of programmed cell death, plays double roles in tumor occurrence and therapeutic mechanisms. Normal cells are activated by many inflammatory molecules secreted during pyrophosis, causing their transformation into tumor cells [19]. In addition, pyroptosis of tumor cells may be a new treatment target [37].

In sarcomas, the problem of how pyroptosis-associated genes relate with each other, and if they are correlated to patients’ survival outcomes, remains unclear. Our study generated a profile characterized by seven pyroptosis-related genes (IGHG2, PODXL2, LRRC17, GABRA3, SCUBE3, HLF and RFLNB) and found that these genes can predict OS in patients. IGHG2 (immunoglobulin heavy constant γ2), a protein coding gene, is associated with some kinds of diseases, for example, immunoglobulin k light chain deficiency. And the related pathways of it are creation of C4 and C2 activators and innate immune system [38]. PODXL2 is a member of the CD34 protein family, whcih is found in endothelial cells and is a ligand for angioselectin, mediating the interaction of white blood cells with the surface of blood vessels [39]. LRRC17 (leucine-rich repeat containing 17), a gene that mainly regulate the osteoblastogenesis process, is primarily expressed in osteoblasts under physiological conditions [40]. α3, GABRA3-γ-aminobutyric acid type A receptor subunit is a protein-coding gene, which is related with diseases including periodic thyrotoxic paralysis and reflex sympathetic dystrophy. The related pathways include ligand-gated ion channel transport and trans-chemical synaptic transport [41]. SCUBE3 (signal peptide, CUB domain and EGF-like domain containing 3) is a protein coding gene. Diseases related with SCUBE3 include facial dysmorphism, short stature and skeletal anomalies with or without cardiac anomalies 2 and bone disease [42]. The gene encodes signal peptides, complement subcomponent C1r/C1s, Uegf, bone morphogenetic protein-1 as well as epidermal growth factor-like domains. Both full-length protein as well as the C-terminal fragment bind transforming growth factor TYPE II receptors to enhance epithelial–mesenchymal transformation and angiogenesis in tumors [43]. HLF, a transcriptional factor, plays an significant regulatory function in various tumors, particularly leukemia and participates in therapy-mediated immunogenic cell death [44]. RFLNB, a member of the Refilin family, is a novel Actin regulatory protein that functions as molecular switches for interconverting the Actin meshwork into bundles. One major trait of this regulatory protein is its short half-life, unique among Actin regulatory proteins [45]. So far, no studies have shown that these seven genes (IGHG2, PODXL2, LRRC17, GABRA3, SCUBE3, HLF and RFLNB) are directly related to pyroptosis in tumor cells. We first proposed these seven genes and analyzed their function and significance in sarcoma.

We investigated the expressions of 33 known pyroptosis-associated genes in sarcomas, analyzed the relationship between each two genes, and investigated their correlation with clinicopathological features. To assess the association between expressions of 33 pyroptosis-related genes and sarcoma subtypes, the NMF algorithm was used to cluster 262 sarcoma samples in the TCGA dataset. About 262 sarcoma patients assigned into two clusters based on consensus clustering matrix. DEG analysis was performed to compare the differential gene expressions of the two clusters and 246 genes were screened. In order to obtain more accurate genes related to cell pyroptosis, we reduced DEG genes to seven genes by univariate Cox regression analysis, random survival forest and LASSO Cox regression dimension reduction. In addition to this, these seven genes, are distributed in randomly chromosome and shown in the Circos plot. With regards to the median score as determined by the risk score formula, 262 patients were assigned into low- and high-risk sub-groups. The overall survival rate in the two groups has obvious differences, patients with the pyroptosis-related genes exhibited a significantly poor overall survival rate, relative to those without pyroptosis-associated genes. In the present study, the IC50 of the 65 drugs in the high-risk group were markedly low, compared with low-risk group, indicating that these drugs may be suitable for high-risk patients, among them, including cisplatin, docetaxel, doxorubicin and gemcitabine. They are currently the main treatment of sarcoma chemotherapy drugs. To determine the underlying mechanisms of different sensitivities of drugs to the high- and low-risk groups, we applied GSEA to our model. Functional analyses revealed that DEGs between low- and high-risk groups was associated with immune and musculoskeletal pathways. We compared the pathways related to infiltration and activation of immune cells in low- and high-risk patient groups and found that the number of infiltrating immune cells as well as activation levels of immune-associated pathways were generally low in high-risk group, relative to the low-risk patient group. Moreover, we also compared the top 30 mutated genes in low- and high-risk patient groups. Among them, mutations of TP53, RB1 as well as MUC16 markedly differed between the groups. In order to further explore the potential target genes of these seven genes in sarcoma. Based on the TRANSFAC database, we obtained transcription factors as well as motifs for seven genes, and mined transcription factors that have been confirmed by studies in sarcoma based on the TF cancer database (Supplementary Table).

Until now, few studies have clearly shown the significance of pyroptosis, particularly its functional mechanisms in sarcomas. We identified seven genes that may have a critical impact on pyroptosis in sarcomas and may serve as regulatory factors. We evaluated the prognostic significance of these pyroptosis-related genes and gave a theoretical basis for subsequent studies. Taken together, our study suggests that pyropwe were unable to confirm whether those previously reported regulatory factors also have similar function in the pyroptosis pathway of sarcoma for lack of data, which deserves further study and experimental verification.

Taken together, our research suggests that pyroptosis is closely associated with sarcoma, which is evidenced by the results that genes associated with or unrelated to pyroptosis are expressed differently in sarcoma tissues. In addition, our score based on risk signature generation of seven pyroptosis-associated genes is an independent risk factor for OS prediction in TCGA cohort. DEGs between low-risk and high-risk patient groups are correlated with tumor immunity and mutations, as well as skeletal muscle pathways. At last, we used qRT-PCR to detect the expression of seven pyroptosis-related genes in sarcoma cells (RH30 and SW982) and human skeletal muscle cells, compared with human skeletal muscle cells, PODXL2, LRRC17, GABRA3, SCUBE3 and RFLNB genes show high expression levels in tumor cells, while IGHG2 and HLF show low expression levels in tumor cells. This result confirms our data analysis.

Our study gives a new genetic feature for prognostic prediction of sarcoma patients, and forms a theoretical basis for the studies on of pyroptosis-associated genes in sarcoma patients in the future.

## Declaration of Humans and/or the use of Human Tissue Samples

Data sets used in this study are all from the public database TCGA, and humans and/or the use of human tissue samples are not involved. All methods were carried out in accordance with relevant guidelines and regulations.

## Supplementary Material

Supplementary Figure S1 and Table S1

Supplementary File

## Data Availability

The following information was supplied regarding data availability: The datasets used in the present study are available from Analyses of Cancer Genome Atlas (TCGA) and the TCGA data in the supplement table

## Abbreviations

DEG: differentially expressed gene
GSEA: gene set enrichment analysis
HLF: hepatic leukemia factor
HSKMC: Human skeletal muscle cells
LRRC17: leucine-rich repeat containing 17
ROC: receiver operating characteristic
TMB: tumor mutation burden
