# Supplementary material for: Novel pyroptosis-associated genes signature for predicting the prognosis of sarcoma and validation
Source: Biosci Rep. 2022 Dec 9;42(12):BSR20221053. doi: 10.1042/BSR20221053 (PMC9742515; doi:10.1042/BSR20221053)
Supplement: Supplementary Figure S1 and Table S1 [file BSR-2022-1053_supp.pdf]

A

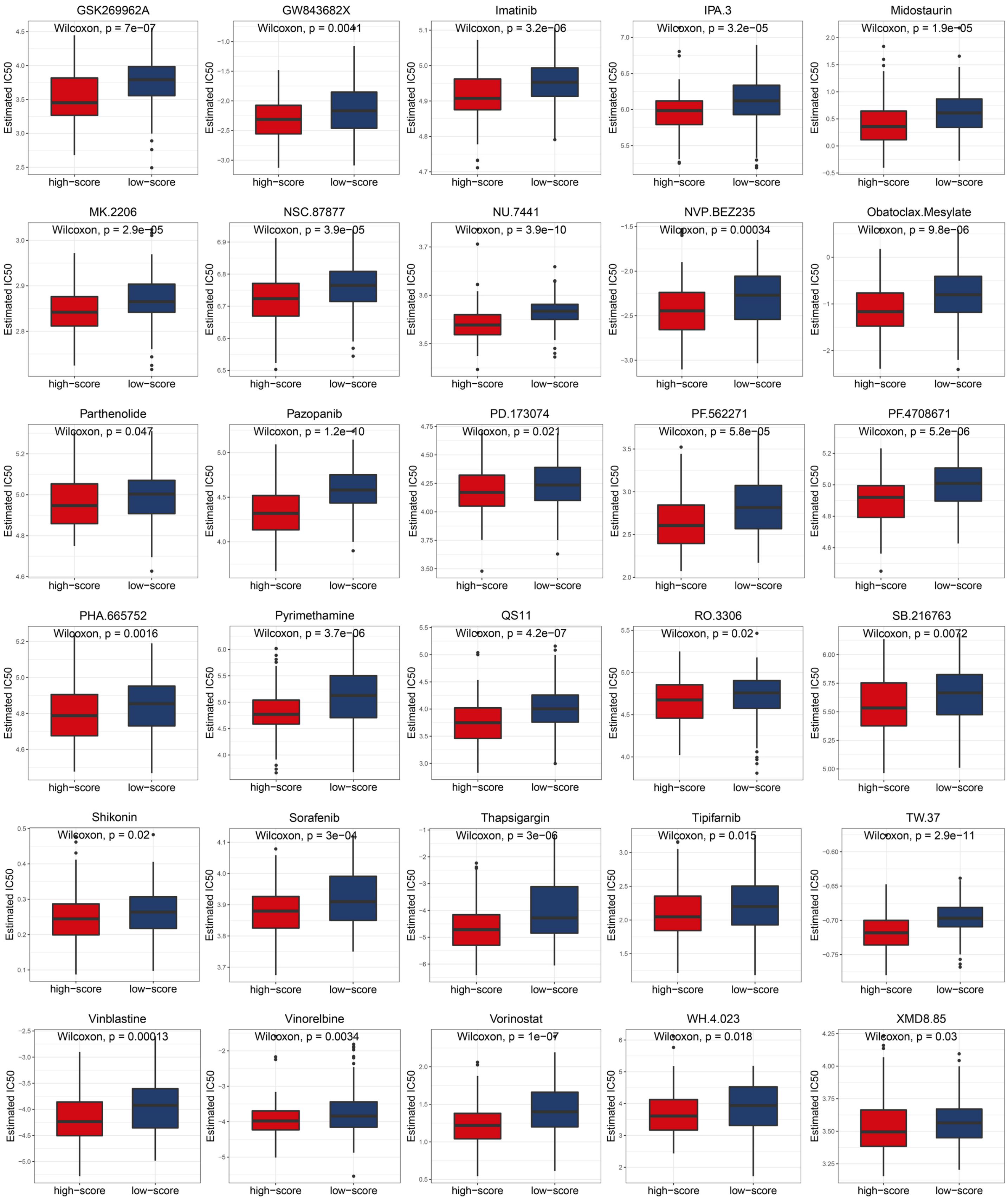
GSK269962A
Wilcoxon, p = 7e−07
GW843682X
Wilcoxon, p = 0.0041
Imatinib
Wilcoxon, p = 3.2e−06
IPA.3
Wilcoxon, p = 3.2e−05
Midostaurin
Wilcoxon, p = 1.9e−05
MK.2206
Wilcoxon, p = 2.9e−05
NSC.87877
Wilcoxon, p = 3.9e−05
NU.7441
Wilcoxon, p = 3.9e−10
NVP.BEZ235
Wilcoxon, p = 0.00034
Obatoclax.Mesylate
Wilcoxon, p = 9.8e−06
Parthenolide
Wilcoxon, p = 0.047
Pazopanib
Wilcoxon, p = 1.2e−10
PD.173074
Wilcoxon, p = 0.021
PF.562271
Wilcoxon, p = 5.8e−05
PF.4708671
Wilcoxon, p = 5.2e−06
PHA.665752
Wilcoxon, p = 0.0016
Pyrimethamine
Wilcoxon, p = 3.7e−06
QS11
Wilcoxon, p = 4.2e−07
RO.3306
Wilcoxon, p = 0.02
SB.216763
Wilcoxon, p = 0.0072
Shikonin
Wilcoxon, p = 0.02
Sorafenib
Wilcoxon, p = 3e−04
Thapsigargin
Wilcoxon, p = 3e−06
Tipifarnib
Wilcoxon, p = 0.015
TW.37
Wilcoxon, p = 2.9e−11
Vinblastine
Wilcoxon, p = 0.00013
Vinorelbine
Wilcoxon, p = 0.0034
Vorinostat
Wilcoxon, p = 1e−07
WH.4.023
Wilcoxon, p = 0.018
XMD8.85
Wilcoxon, p = 0.03
Estimated IC50
high−score
low−score

B

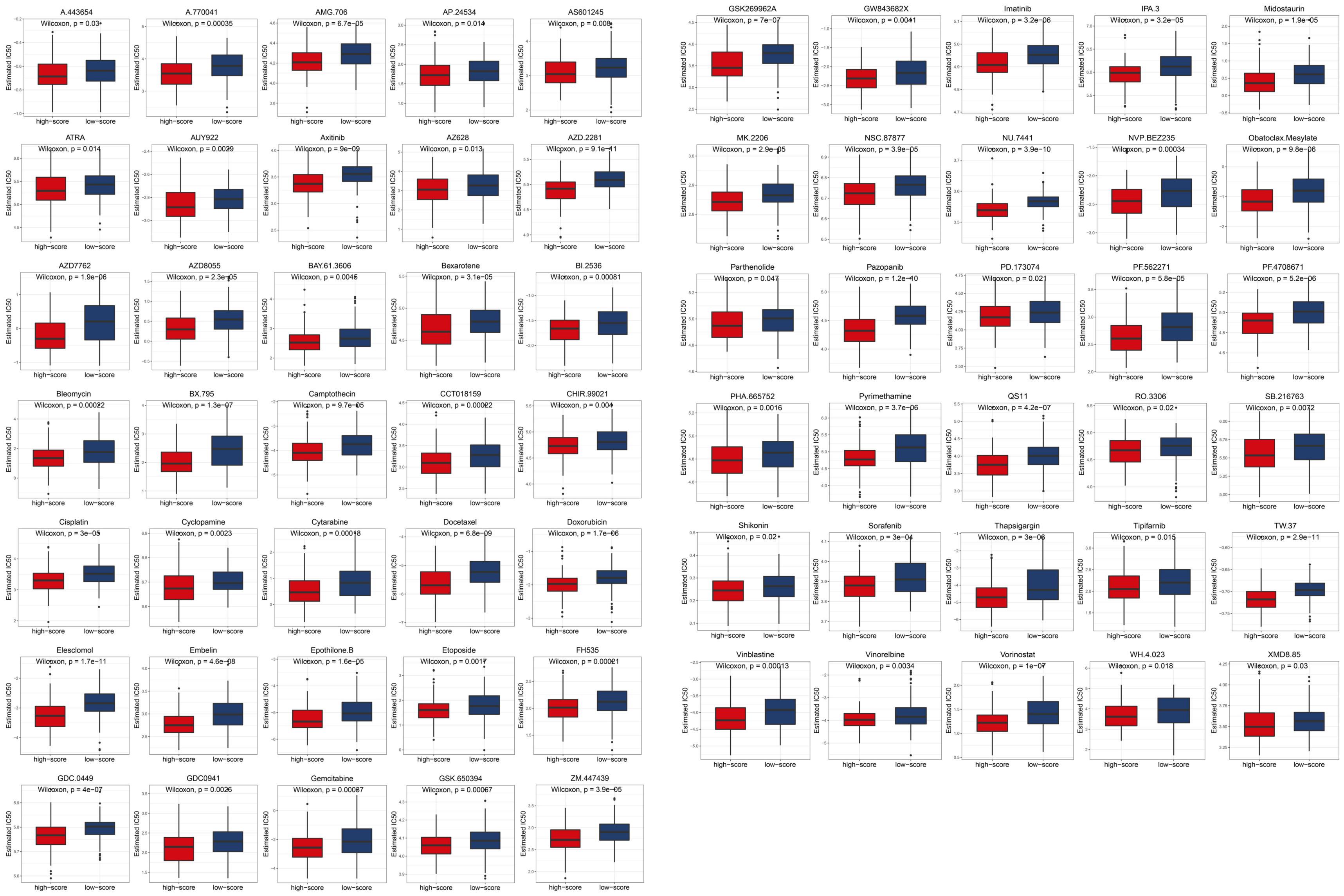

A.443654
Wilcoxon, p = 0.03
A.770041
Wilcoxon, p = 0.00035
AMG.706
Wilcoxon, p = 6.7e−05
AP.24534
Wilcoxon, p = 0.014
AS601245
Wilcoxon, p = 0.008
ATRA
Wilcoxon, p = 0.014
AUY922
Wilcoxon, p = 0.0029
Axitinib
Wilcoxon, p = 9e−09
AZ628
Wilcoxon, p = 0.013
AZD.2281
Wilcoxon, p = 9.1e−11
AZD7762
Wilcoxon, p = 1.9e−06
AZD8055
Wilcoxon, p = 2.3e−05
BAY.61.3606
Wilcoxon, p = 0.0045
Bexarotene
Wilcoxon, p = 3.1e−05
BI.2536
Wilcoxon, p = 0.00081
Bleomycin
Wilcoxon, p = 0.00022
BX.795
Wilcoxon, p = 1.3e−07
Camptothecin
Wilcoxon, p = 9.7e−05
CCT018159
Wilcoxon, p = 0.00022
CHIR.99021
Wilcoxon, p = 0.004
Cisplatin
Wilcoxon, p = 3e−05
Cyclopamine
Wilcoxon, p = 0.0023
Cytarabine
Wilcoxon, p = 0.00018
Docetaxel
Wilcoxon, p = 6.8e−09
Doxorubicin
Wilcoxon, p = 1.7e−06
Elesclomol
Wilcoxon, p = 1.7e−11
Embelin
Wilcoxon, p = 4.6e−08
Epothilone.B
Wilcoxon, p = 1.6e−05
Etoposide
Wilcoxon, p = 0.0017
FH535
Wilcoxon, p = 0.00021
GDC.0449
Wilcoxon, p = 4e−07
GDC0941
Wilcoxon, p = 0.0026
Gemcitabine
Wilcoxon, p = 0.00087
GSK.650394
Wilcoxon, p = 0.00067
ZM.447439
Wilcoxon, p = 3.9e−05
GSK269962A
Wilcoxon, p = 7e−07
GW843682X
Wilcoxon, p = 0.0041
Imatinib
Wilcoxon, p = 3.2e−06
IPA.3
Wilcoxon, p = 3.2e−05
Midostaurin
Wilcoxon, p = 1.9e−05
MK.2206
Wilcoxon, p = 2.9e−05
NSC.87877
Wilcoxon, p = 3.9e−05
NU.7441
Wilcoxon, p = 3.9e−10
NVP.BEZ235
Wilcoxon, p = 0.00034
Obatoclax.Mesylate
Wilcoxon, p = 9.8e−06
Parthenolide
Wilcoxon, p = 0.047
Pazopanib
Wilcoxon, p = 1.2e−10
PD.173074
Wilcoxon, p = 0.021
PF.562271
Wilcoxon, p = 5.8e−05
PF.4708671
Wilcoxon, p = 5.2e−06
PHA.665752
Wilcoxon, p = 0.0016
Pyrimethamine
Wilcoxon, p = 3.7e−06
QS11
Wilcoxon, p = 4.2e−07
RO.3306
Wilcoxon, p = 0.02
SB.216763
Wilcoxon, p = 0.0072
Shikonin
Wilcoxon, p = 0.02
Sorafenib
Wilcoxon, p = 3e−04
Thapsigargin
Wilcoxon, p = 3e−06
Tipifarnib
Wilcoxon, p = 0.015
TW.37
Wilcoxon, p = 2.9e−11
Vinblastine
Wilcoxon, p = 0.00013
Vinorelbine
Wilcoxon, p = 0.0034
Vorinostat
Wilcoxon, p = 1e−07
WH.4.023
Wilcoxon, p = 0.018
XMD8.85
Wilcoxon, p = 0.03
Estimated IC50
high-score
low-score

Supplementary Figure :   IC50 values of all 79 drugs. A. 14 drugs with low drug sensitivity of the risk group. B. 65 drugs with high drug sensitivity of the risk group.

pmid	TF	characteristics	gene	regulation_type	hallmark	original_text	title	Motif	Strand	Location	p.value	Match.Sequence	Gene
28643791	PAX7	high expression; tageted by	EWSR1 fusion protein	positive	Ewing sarcoma	Here, using analyses of published whole-genome gene expression microarray data, we identify PAX7 as a gene with significantly increased expression in Ewing sarcoma in comparison to CIC-DUX4 round cell sarcoma.Exploring the mechanism of PAX7 expression in Ewing sarcoma using curated RNA- and ChIP-sequencing data, we demonstrate that the EWSR1 fusion protein is required for PAX7 expression in Ewing sarcoma and identify a candidate EWSR1-FLI1-bound PAX7 enhancer that coincides with both a consensus GGAA repeat-containing binding site and a peak of regulatory H3K27 acetylation.	EWSR1 fusion proteins mediate PAX7 expression in Ewing sarcoma.	V_PAX7_01_M01339	-	102557208-102557224	5.00E-06	TCAAATAATTAGAAAAA	LRRC17
28008375	SMAD3	targeted by	EWSR1/FLI	negative	N/A	SMAD3 were up-regulated and FLI1, MYB, E2F1, ETS2, WT1 were down-regulated with more than half of their targets were down-regulated after EWSR1/FLI1 knockdown.	Knockdown of EWSR1/FLI1 expression alters the transcriptome of Ewing sarcoma cells in vitro.	V_SMAD3_Q6_01_M01888	-	127348889-127348901	7.00E-06	AGGCAGACACATC	PODXL2
28008375	MYB	targeted by	EWSR1/FLI	positive	N/A	SMAD3 were up-regulated and FLI1, MYB, E2F1, ETS2, WT1 were down-regulated with more than half of their targets were down-regulated after EWSR1/FLI1 knockdown.	Knockdown of EWSR1/FLI1 expression alters the transcriptome of Ewing sarcoma cells in vitro.	V_MYB_Q3_M00773	+	102557023-102557033	2.00E-06	AGTGTCAGTTG	LRRC17
29773426	SOX2	correlate with	H3K27me3	N/A	synovial sarcoma	H3K27me3 immunohistochemistry of the synovial sarcoma cases revealed a high statistically significant correlation between SOX2 and H3K27me3 expression (p_<_0,0005, Chi square test). Six SOX2 positive synovial sarcoma cases were analyzed by FISH using a SOX2/CEN3 dual color FISH probe. None of these cases revealed an amplification of the SOX2 gene.	Stem cell transcription factor SOX2 in synovial sarcoma and other soft tissue tumors.	V_SOX2_Q6_M01272	+	102553198-102553213	1.00E-05	ATTCTCTTTGTTTGTG	LRRC17
7828148	CHOP	amplification; high expression	N/A	N/A	N/A	We have now found CHOP amplification in two sarcoma cell lines with previously reported amplification of the nearby GLI gene. High constitutive expression levels of CHOP were observed in tumors with gene amplification, but also in some other samples. MDM2 and CHOP were co-amplified in two of these, whereas the two osteosarcomas had amplified CHOP but not MDM2. CHOP was amplified in both cell lines with GLI amplification, and MDM2 only in one.	The protooncogene CHOP/GADD153, involved in growth arrest and DNA damage response, is amplified in a subset of human sarcomas.	V_CHOP_01_M00249	+	102557059-102557071	7.00E-06	TTGTGCAATACTC	LRRC17
10574952	ATF1	high expression	N/A	N/A	cell viability; clear cell sarcoma	The level of EWS/ATF1 expression was found to be significantly higher in primary tumor tissue than in SU-CCS-1 cells or in 293T cells following introduction of an EWS/ATF1 expression vector. These studies demonstrate a direct role for the EWS/ATF1 fusion protein in maintaining tumor cell viability of Clear Cell sarcoma and indicate that intracellular antibodies may be used to achieve a phenotypic knockout of tumor-related proteins as a method to explore their function.	Tumor cell viability in clear cell sarcoma requires DNA binding activity of the EWS/ATF1 fusion protein.	V_ATF1_Q6_M00691	+	53342311-53342321	1.00E-06	CCCTGACGTCA	HLF
10574952	ATF1	high expression	N/A	N/A	cell viability; clear cell sarcoma	The level of EWS/ATF1 expression was found to be significantly higher in primary tumor tissue than in SU-CCS-1 cells or in 293T cells following introduction of an EWS/ATF1 expression vector. These studies demonstrate a direct role for the EWS/ATF1 fusion protein in maintaining tumor cell viability of Clear Cell sarcoma and indicate that intracellular antibodies may be used to achieve a phenotypic knockout of tumor-related proteins as a method to explore their function.	Tumor cell viability in clear cell sarcoma requires DNA binding activity of the EWS/ATF1 fusion protein.	V_ATF1_04_M02842	+	102553731-102553744	7.00E-06	CTATGACAAAGAAA	LRRC17
20514024	EGR1	regulate	PTEN	positive; promoter binding	cell death; synovial sarcoma	Moreover, we find that under these conditions phosphatase and tensin homolog deleted in chromosome 10 (PTEN) is upregulated and this occurs through direct binding of EGR1 to an element upstream of the PTEN promoter. Using a combination of gain- and loss-of-function approaches, we show that EGR1 modulation of PTEN contributes to HDAC inhibitor-induced apoptosis in synovial sarcoma. Finally, restoration of EGR1 or PTEN expression is sufficient to induce synovial sarcoma cell death.	EGR1 reactivation by histone deacetylase inhibitors promotes synovial sarcoma cell death through the PTEN tumor suppressor.	V_EGR1_Q6_M01873	-	53342277-53342286	4.00E-06	GCGGGGGCGG	HLF
20514024	EGR1	regulate	PTEN	positive; promoter binding	cell death; synovial sarcoma	Moreover, we find that under these conditions phosphatase and tensin homolog deleted in chromosome 10 (PTEN) is upregulated and this occurs through direct binding of EGR1 to an element upstream of the PTEN promoter. Using a combination of gain- and loss-of-function approaches, we show that EGR1 modulation of PTEN contributes to HDAC inhibitor-induced apoptosis in synovial sarcoma. Finally, restoration of EGR1 or PTEN expression is sufficient to induce synovial sarcoma cell death.	EGR1 reactivation by histone deacetylase inhibitors promotes synovial sarcoma cell death through the PTEN tumor suppressor.	V_EGR1_06_M02744	+	53342276-53342289	1.00E-06	CCCGCCCCCGCACG	HLF
24415532	REST	targeted by	EWS-FLI1	positive	tumor growth	Inhibition of EWS-FLI-1 using small interfering RNA decreased REST expression in human Ewing sarcoma cells. Inhibition of REST did not affect EWS-FLI-1, but significantly suppressed tumor growth in vivo, reduced the tumor vessel pericyte markers - smooth muscle actin (SMA) and desmin, increased hypoxia and apoptosis in tumor tissues, and decreased the expression of delta-like ligand 4 (DLL4) and Hes1.	EWS-FLI-1 regulates the neuronal repressor gene REST, which controls Ewing sarcoma growth and vascular morphology.	V_REST_01_M01256	+	35182303-35182324	1.00E-06	CCTGGGGGCTGTCCTGGCTGCT	SCUBE3
24415532	REST	targeted by	EWS-FLI1	positive	tumor growth	Inhibition of EWS-FLI-1 using small interfering RNA decreased REST expression in human Ewing sarcoma cells. Inhibition of REST did not affect EWS-FLI-1, but significantly suppressed tumor growth in vivo, reduced the tumor vessel pericyte markers - smooth muscle actin (SMA) and desmin, increased hypoxia and apoptosis in tumor tissues, and decreased the expression of delta-like ligand 4 (DLL4) and Hes1.	EWS-FLI-1 regulates the neuronal repressor gene REST, which controls Ewing sarcoma growth and vascular morphology.	V_REST_02_M02256	-	35182307-35182327	7.00E-06	CCCAGCAGCCAGGACAGCCCC	SCUBE3
24415532	REST	regulate	SMA; desmin; DLL4; Hes1	positive	N/A	Inhibition of EWS-FLI-1 using small interfering RNA decreased REST expression in human Ewing sarcoma cells. Inhibition of REST did not affect EWS-FLI-1, but significantly suppressed tumor growth in vivo, reduced the tumor vessel pericyte markers - smooth muscle actin (SMA) and desmin, increased hypoxia and apoptosis in tumor tissues, and decreased the expression of delta-like ligand 4 (DLL4) and Hes1.	EWS-FLI-1 regulates the neuronal repressor gene REST, which controls Ewing sarcoma growth and vascular morphology.	V_REST_01_M01256	+	35182303-35182324	1.00E-06	CCTGGGGGCTGTCCTGGCTGCT	SCUBE3
24415532	REST	regulate	SMA; desmin; DLL4; Hes1	positive	N/A	Inhibition of EWS-FLI-1 using small interfering RNA decreased REST expression in human Ewing sarcoma cells. Inhibition of REST did not affect EWS-FLI-1, but significantly suppressed tumor growth in vivo, reduced the tumor vessel pericyte markers - smooth muscle actin (SMA) and desmin, increased hypoxia and apoptosis in tumor tissues, and decreased the expression of delta-like ligand 4 (DLL4) and Hes1.	EWS-FLI-1 regulates the neuronal repressor gene REST, which controls Ewing sarcoma growth and vascular morphology.	V_REST_02_M02256	-	35182307-35182327	7.00E-06	CCCAGCAGCCAGGACAGCCCC	SCUBE3
24069508	REST	interact with	EWS	N/A	neuronal phenotype development; oncogenic transformation; Ewing sarcoma	Co-immunoprecipitation analysis demonstrated that EWS interacts directly with REST. Genome-wide binding analysis showed that EWS binds chromatin at or near NRSE. Furthermore, functional studies revealed that both EWS and REST inhibit neuronal phenotype development and oncogenic transformation in Ewing sarcoma cells.	EWS and RE1-Silencing Transcription Factor Inhibit Neuronal Phenotype Development and Oncogenic Transformation in Ewing Sarcoma.	V_REST_01_M01256	+	35182303-35182324	1.00E-06	CCTGGGGGCTGTCCTGGCTGCT	SCUBE3
24069508	REST	interact with	EWS	N/A	neuronal phenotype development; oncogenic transformation; Ewing sarcoma	Co-immunoprecipitation analysis demonstrated that EWS interacts directly with REST. Genome-wide binding analysis showed that EWS binds chromatin at or near NRSE. Furthermore, functional studies revealed that both EWS and REST inhibit neuronal phenotype development and oncogenic transformation in Ewing sarcoma cells.	EWS and RE1-Silencing Transcription Factor Inhibit Neuronal Phenotype Development and Oncogenic Transformation in Ewing Sarcoma.	V_REST_02_M02256	-	35182307-35182327	7.00E-06	CCCAGCAGCCAGGACAGCCCC	SCUBE3
24043308	GLI1	regulate	KRT17	positive	cellular adhesion; oncogenic transformation	In this work, we identify keratin 17 (KRT17) as a direct downstream target gene upregulated by GLI1. We demonstrate that KRT17 regulates cellular adhesion by activating AKT/PKB (protein kinase B) signaling. In addition, KRT17 is necessary for oncogenic transformation in Ewing sarcoma and accounts for much of the GLI1-mediated transformation function but via a mechanism independent of AKT signaling.	A novel role for keratin 17 in coordinating oncogenic transformation and cellular adhesion in Ewing sarcoma.	V_GLI1_01_M01702	-	102556953-102556963	8.00E-06	GGCCACCCAAG	LRRC17
11973649	ETS1	targeted by	PARP-1	negative	N/A	Previously, we cloned the PARP gene promoter region from EWS cells, showed that it contained multiple ETS-binding sites and demonstrated a positive regulation of PARP by ETS1.Results show that stable down-regulation of ETS1 increases the resistance of EWS cells to various genotoxic agents, whereas down-regulation of EWS/FLI-1 has pro-apoptotic effects. Because down-regulation EWS/FLI-1 does not dramatically change PARP levels, these results suggest a direct effect for EWS/FLI-1 in the DNA damage response of EWS cells.	Differential regulation of the response to DNA damage in Ewing's sarcoma cells by ETS1 and EWS/FLI-1.	V_ETS1_B_M00339	-	102549332-102549346	0	CCAGGAAGTGGTTAC	LRRC17
30219084	ATF1	EWSR1-ATF1 fusion	N/A	N/A	N/A	These cases most likely had EWSR1-ATF1 and EWSR1-CREB1 fusions, respectively. RT-PCR was performed in 8 available cases, including 6 CCSSTs and 2 CCSLGTs. All CCSSTs showed EWSR1-ATF1 fusions. Among the 2 CCSLGT cases, one had EWSR1-ATF1 fusion and the other had EWSR1-CREB1 fusion.	Detection of specific gene rearrangements by fluorescence in situ hybridization in 16 cases of clear cell sarcoma of soft tissue and 6 cases of clear cell sarcoma-like gastrointestinal tumor.	V_ATF1_Q6_M00691	+	53342311-53342321	1.00E-06	CCCTGACGTCA	HLF
30219084	ATF1	EWSR1-ATF1 fusion	N/A	N/A	N/A	These cases most likely had EWSR1-ATF1 and EWSR1-CREB1 fusions, respectively. RT-PCR was performed in 8 available cases, including 6 CCSSTs and 2 CCSLGTs. All CCSSTs showed EWSR1-ATF1 fusions. Among the 2 CCSLGT cases, one had EWSR1-ATF1 fusion and the other had EWSR1-CREB1 fusion.	Detection of specific gene rearrangements by fluorescence in situ hybridization in 16 cases of clear cell sarcoma of soft tissue and 6 cases of clear cell sarcoma-like gastrointestinal tumor.	V_ATF1_04_M02842	+	102553731-102553744	7.00E-06	CTATGACAAAGAAA	LRRC17
16463269	STAT3	N/A	N/A	N/A	Ewing sarcoma family of tumours	In conclusion, STAT3 activation is present in approximately half of ESFT and correlates with clinical features. The role of STAT3 activation in ESFT pathogenesis seems to be independent of the type of EWS/Ets translocation.	STAT3 is activated in a subset of the Ewing sarcoma family of tumours.	V_STAT3_03_M01595	-	127351434-127351449	4.00E-06	CATTCCAGGAAGAAAA	PODXL2
23185447	ETS	regulate	KCNN2	negative	Ewing\'s sarcoma	Conversely, KCNN2 was found underexpressed in ESFT relative to ARMS, suggesting that the EWSR1-ETS oncoprotein may have the opposite effect of ERG rearrangements in PCa.	Potential downstream target genes of aberrant ETS transcription factors are differentially affected in Ewing's sarcoma and prostate carcinoma.	V_ETS_B_M00340	-	102549334-102549347	6.00E-06	GCCAGGAAGTGGTT	LRRC17
23185447	ETS	regulate	KCNN2	negative	Ewing\'s sarcoma	Conversely, KCNN2 was found underexpressed in ESFT relative to ARMS, suggesting that the EWSR1-ETS oncoprotein may have the opposite effect of ERG rearrangements in PCa.	Potential downstream target genes of aberrant ETS transcription factors are differentially affected in Ewing's sarcoma and prostate carcinoma.	V_ETS_Q4_M00771	+	102549334-102549345	0	AACCACTTCCTG	LRRC17
23995784	FOXO1	targeted by	EWS-FLI1	negative; EWS-FLI1-suppressed regulator	decrease in ES tumor growth	In addition to FOXO1 regulation by direct promoter binding of EWS-FLI1, its subcellular localization and activity is regulated by cyclin-dependent kinase 2- and AKT-mediated phosphorylation downstream of EWS-FLI1. Restoration of nuclear FOXO1 expression in ES cells impaired proliferation and significantly reduced clonogenicity. Gene-expression profiling revealed a significant overlap between EWS-FLI1-repressed and FOXO1-activated genes.	Suppression of FOXO1 is responsible for a growth regulatory repressive transcriptional sub-signature of EWS-FLI1 in Ewing sarcoma.	V_FOXO1_Q5_M01216	-	102557196-102557204	8.00E-06	AAAAACAAG	LRRC17
23995784	FOXO1	targeted by	EWS-FLI1	negative; EWS-FLI1-suppressed regulator	decrease in ES tumor growth	In addition to FOXO1 regulation by direct promoter binding of EWS-FLI1, its subcellular localization and activity is regulated by cyclin-dependent kinase 2- and AKT-mediated phosphorylation downstream of EWS-FLI1. Restoration of nuclear FOXO1 expression in ES cells impaired proliferation and significantly reduced clonogenicity. Gene-expression profiling revealed a significant overlap between EWS-FLI1-repressed and FOXO1-activated genes.	Suppression of FOXO1 is responsible for a growth regulatory repressive transcriptional sub-signature of EWS-FLI1 in Ewing sarcoma.	V_FOXO1_01_M00473	-	35185882-35185891	3.00E-06	AAAAAACAAT	SCUBE3

Supplementary Table : transcription factor motifs of seven genes.
